# Supplementary material for: Characterization of the 1-Deoxy-D-xylulose 5-Phosphate synthase Genes in Toona ciliata Suggests Their Role in Insect Defense
Source: Int J Mol Sci. 2023 Jan 25;24(3):2339. doi: 10.3390/ijms24032339 (PMC9917211; doi:10.3390/ijms24032339)
Supplement: Supplementary file 1 [file ijms-24-02339-s001.zip › Supplemental Tables.pdf]

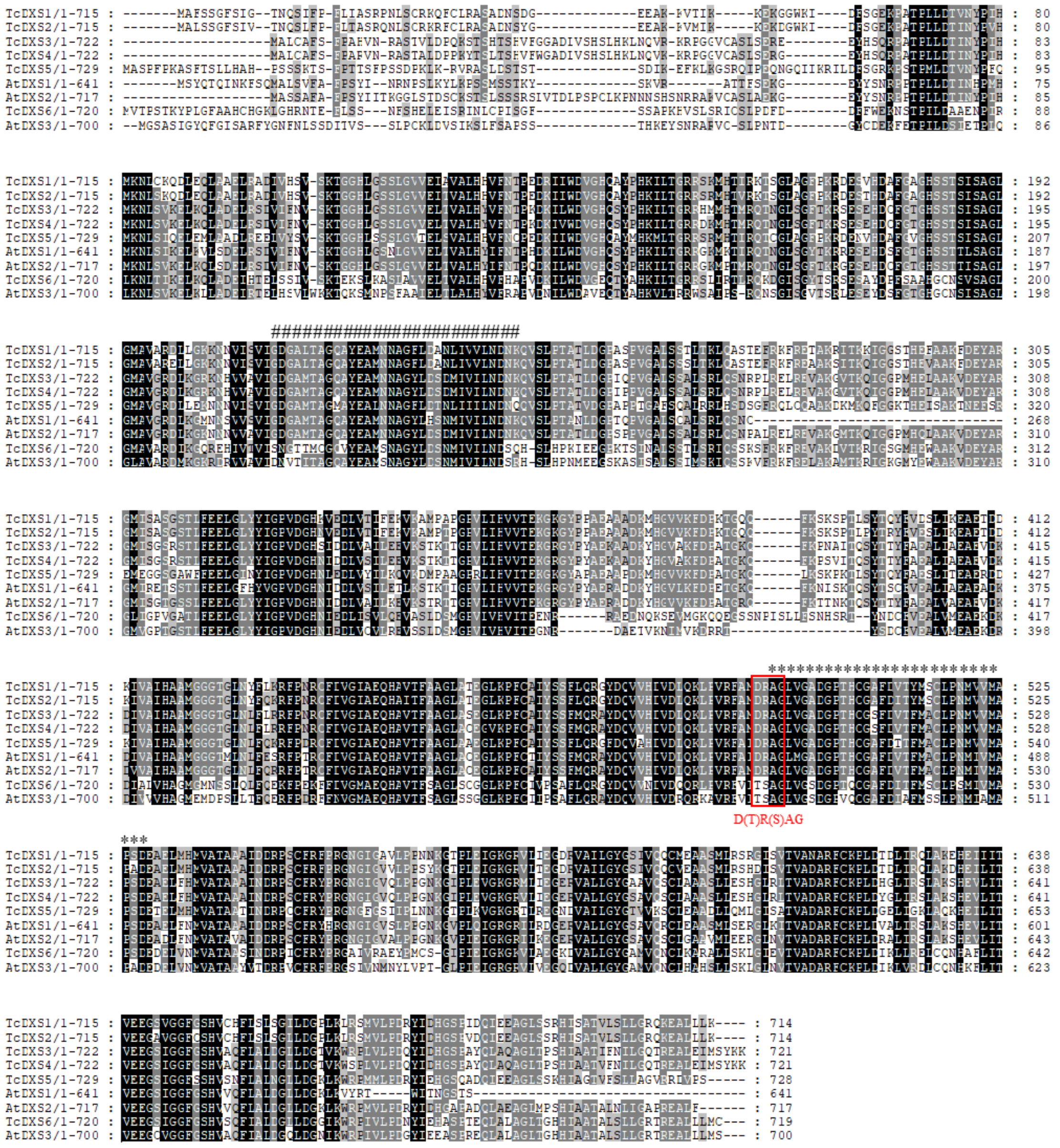

Figure S1. Multiple sequence alignment of the protein sequences of TcDXSs and AtDXSs obtained from *T. ciliata* (this study) and *A. thaliana*. # and * indicate the regions corresponding to the Transket-pyr binding site and Transketolase-C binding site, respectively. Red square box indicates the special regions ‘D(T)R(S)AG’ of DXSs.

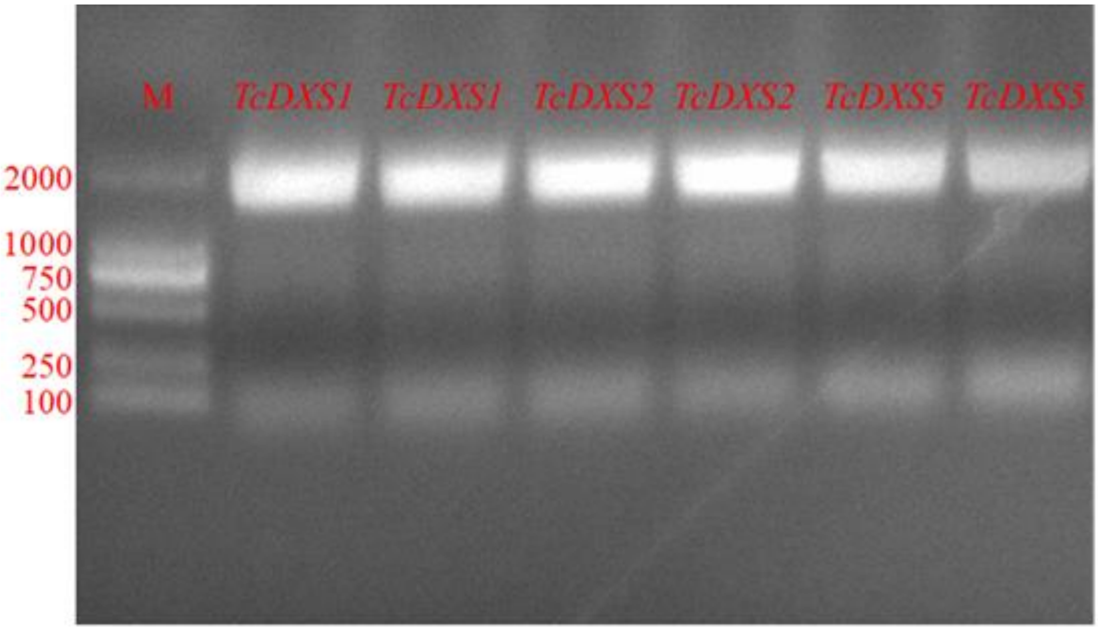

Fig. S2 Amplification products of TcDXS1 TcDXS2 and TcDXS5 coding sequence

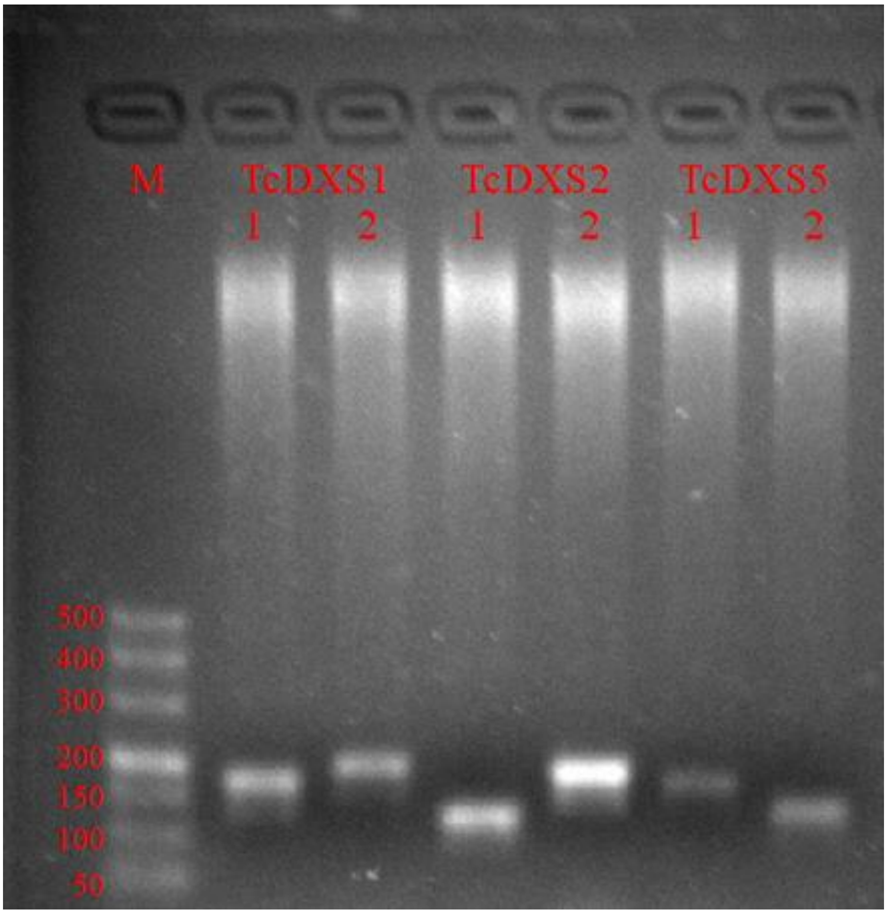

Fig. S3 qPCR primer analysis of TcDXS1 TcDXS2 and TcDXS5 cDNA

Table S1 Protein sequence of *DXSs*

**Species**	**Gene name**	**Gene ID**	**Gene Bank**	**Protein sequence**	**Database**
Arabidopsis thaliana	*AtDXS1*	At4g15560		MASSAFAFPSYIITKGGLSTDSCKSTSLSSSRSLVTDLPSPCLKPNNNSHSNRRAKVCASLAEKGE<br>YYSNRPPTPLLDTINYPIHMKNLSVKELKQLSDELRSDVIFNVSKTGGHLGSSLGVVELTVALHY<br>IFNTPQDKILWDVGHQSYPHKILTGRRGKMPTMRQTNGLSGFTKRGESEHDCFGTGHSSTTISA<br>GLGMAVGRDLKGKNNNVVAVIGDGAMTAGQAYEAMNNAGYLDSDMIVILNDNKQVSLPTAT<br>LDGPSPPVGALSSALSRLQSNPALRELREVAKGMTKQIGGPMHQLAAKVDEYARGMISGTGSSL<br>FEELGLYYIGPVDGHNIDDLVAILKEVKSTRTTGPVLIHVVTEKGRGYPYAERADDKYHGVVKF<br>DPATGRQFKTTNKTQSYTTYFAEALVAEAEVDKDVVAIHAAMGGGTGLNLFQRRFPTRCFDVG<br>IAEQHAVTFAAGLACEGLKPFCAIYSSFMQRAYDQVVHDVDLQKLPVRFAMDRAGLVGADGP<br>THCGAFDVTFMACLPNMIVMAPSDEADLFNMVATAVAIDDRPSCFRYPRGNGIGVALPPGNKG<br>VPIEIGKGRILKEGERVALLGYGSAVQSCLGAAVMLEERGLNVTVADARFCKPLDRALIRSLAK<br>SHEVLITVEEGSIGGFGSHVVQFLALDGLLDGKLKWRPMVLPDRYIDHGAPADQLAEAGLMPS<br>HIAATALNLIGAPREALF	TAIR
Arabidopsis thaliana	*AtDXS2*	At3g21500		MSYQTQINKFSQMALSVFAFPSYINRNPSLKYLKPSSMSSTKYSKVRATTFSEKGEYYSNRPPTP<br>LLDTINHPMHMKNLSIKELKVLSDELRSDVIFNVSKTGGHLGSNLGVVELTVALHYIFNTPHDKI<br>LWDVGHQSYPHKILTGRRGKMKTIRQTNGLSGYTKRRESEHDSFGTGHSSTTLSAGLGMAVGR<br>DLKGMNNSVVSVIGDGAMTAGQAYEAMNNAGYLHSNMIVILNDNKQVSLPTANLDGPTQPVG<br>ALSCALSRLQSNCGMIRETSSTLFEELGFHYVGPVDGHNIDDLVSILETLKSTKTIGPVLIHVVTE<br>KGRGYPYAERADDKYHGVLKFDPETGKQFKNISKTQSYTSCFVEALIAEAEADKDIVAIHAAM<br>GGGTMLNLFESRFPTRCFDVGIAEQHAVTFAAGLACEGLKPFCTIYSSFMQRAYDQVVHDVDL<br>QKLPVRFAIDRAGLMGADGPTHCGAFDVTFMACLPNMIVMAPSDEAELFNMVATAAAIDDRPS<br>CFRYHRGNGIGVSLPPGNKGVPLQIGRGRILRDGERVALLGYGSAVQRCLEAASMLSERGLKIT<br>VADARFCKPLDVALIRSLAKSHEVLITVEEGSIGGFGSHVVQFLALDGLLDGKLKVYRTWITNG<br>STS	TAIR
Arabidopsis thaliana	*AtDXS3*	At5g11380		MGSASIGYQFGISARFYGNFNLSSDITVSSLPCKLDVSIKSLFSAPSSTHKEYSNRARVCSLPNTD<br>GYCDEKFETPILDSIETPLQLKNLSVKELKLLADEIRTELHSVLWKKTQKSMNPSFAAIELTLALH<br>YVFRAPVDNILWDAVEQTYAHKVLTRRWSAIPSRQNSGISGVTSRLESEYDSFGTGHGCNSISA<br>GLGLAVARDMKGKRDRVVAVIDNVTITAGQAYEAMSNAGYLDSNMIVILNDSRHSLHPNMEE<br>GSKASISALSSIMSKIQSSKVFRKFRELAKAMTKRIGKGMYEWAAKVDEYARGMVGPTGSTLFE	TAIR

Species	Gene name	Gene ID	Gene Bank	Protein sequence	Database
				ELGLYYIGPVDGHNIEDLVCVLREVSSLDSMGPVLVHVITEGNRDAETVKNIMVKDRRTYSDCF<br>VEALVMEAEKDRDIVVVHAGMEMDPSLLTFQERFPDRFFNVGMAEQHAVTFSAGLSSGGLKPF<br>CIIPSAFLQRAYDQVVHDVDRQRKAVRFVITSAGLVGSDGPVQCGAFDIAFMSSLPNMIAMAPA<br>DEDELVNMVATAAYVTDRPVCFRFPRGSIVNMNYLVPTGLPIEIGRGRVLVEGQDVALLGYGA<br>MVQNCLHAHSLLSKLGLNVTVADARFCKPLDIKLVRDLCQNHKFLITVEEGCVGGFGSHVAQFI<br>ALDGQLDGNIKWRPIVLPDGYIEEASPREQLALAGLTGHHIAATALSLLGRTREALLLMS	
Ginkgo biloba	*GbDXS1*		AAS89341.1	MAMLMASTAILRPNSKQSPSMFFGLLPCGLRQQIKPGNLEFEQSRRKVGKAYAAALSEKGEYYS<br>EKPPTPLLDTINYPIHMKNLSLRELKQLADELRADLIFDVSRTGGHLGSSLGVIELTVAIHYVFNA<br>PEDKIFWDVGHQAYPHKILTGRRDKMPTLRQTNGLSGFTKRSESEYDSFGAGHSSTSISAGLGM<br>AVGRDLKGRNNHVISVIGDGAMTAGQAYEAMNNAGYLDSNMIVILNDNKQVSLPTANLDGPIP<br>PVGALSSALSRLQSSRPLRELREVAKGVTKQLGGSMHELAAKVDEYARGMISGSRSTLFEEMGL<br>YYIGPVDGHNIDDLVTILREVKATHTTGPVLIHVVTEKGRGYPYAERAADKYHGVVKFDPATG<br>KQFKGKAQTQSYTTYFAEALIAEAEVDKNIVAIHAAMGGGTGLNMFLKRFPSRCFDVGIAEQH<br>AVTFAAGLACEGLKPFCAIYSSFLQRAYDQVVHDVDVDLQKLPVRFAMDRAGLVGADGPTHC<br>GAFDVTYLSCLPNMVVMAPSDEAELFHMVATAAAIDDRPSCFRYPRGNGIGVQLPIGNKGVPLE<br>VGKGRILAEGDRVALLGYGTVVQNCLAAGALLEEQGLSLTVADARFCKPLDRDLIRSLAREHE<br>VLITVEEGSIGGFGSHVSQFLSLDGLLDGKLKWRPLVLPDRYIEHGAPIDQMADAGLTASHIAAS<br>VLNILGRTRDAPQIMS	NCBI
Ginkgo biloba	*GbDXS2*		AAR95699.1	MAASSMQAVSFPQVDCQSHFHSIPELGSFSGLRKILPATTAYNRSAATKKKGCALYTKANANNS<br>DGGESRKIVTAGVEVGGKIDFSGEKPATPLLDTINYPIHMKNLSVEDLRQLASELRAEIVFGVSK<br>TGGHLGASLGVVDLTVALHHVFNTPEDRIIWDVGHQAYPHKILTGRRSRMHTIRQTSGLAGFPK<br>RDESEHDAFGAGHSSTSISAGLGMAVGRDLLGKRNHVVAVIGDGAMTAGQAYEAMNNSGYLD<br>SNMIIILNDNKQVSLPTATLDGAAPPVGALSSALTKPQSSKKLRKLREAAKSLTKQIGGQTHEIAS<br>KVDEYARGMISPAGSSLFEELGLYYIGPVDGHNMEDMVTILDKVKSMPAPGPVLIHLVTDKGK<br>GYPPAEKAADKLHGVVNFDPATGKQFKSKSSTLSYTQYFADALIAEAEEDSKIVAIHAAMGGGT<br>GLNYFQKRFPDRCFDVGIAEQHAVTFAAGMATEGLKPFCAIYSTFLQRGYDQVVHDVDLQKLP<br>VRFAMDRAGLVGADGPTHCGAFDITYMACLPNMVVMAPSDEAELMHMIATAAAIDDRPSCFR<br>FPRGNGVGVPLPPGNRGIPLEIGKGCILQEGSRVAILGCGAIVQNCVAARMLLKEHDISITIADAR<br>FCKPLDGDLIRQLAKEHEILVTVEEGAIGGFGSHVPHFLALNGLLDGNLKWRAMVLPDRYIEHG<br>SPKDQIEEAGLSSRHIASTVMSPLGRPHDALQLSN	NCBI

Species	Gene name	Gene ID	Gene Bank	Protein sequence	Database
Hevea brasiliensis	*HbDXS1*		AAS94123.1	MALSACSFPAHVNRNTISDLQKYSYVSSHFLSRKNPLAQSLHRLNQAKSKRRPERVCASLSEREEYHSQRPPTPLLDTINYPIHMKNLSIKELKQLADELRSDVIFNVSGTGGHLGSSLGVVELTVALHYVFNAPQDKILWDVGHQSYPHKILTGRRDKMHTIRQTNGLSGFTKRSESEYDCFGTGHSSTTISAGLGMAVGRDLKGRKNNVVAVIGDGAMTAGQAYEAMNNAGYLDSDMIVILNDNKQVSLPTATLDGPIPPVGALSSALSRLQSNRPLRELREVAKGVTKRIGGSMHELAAKVDEYARGMISGSGSTLFEELGLYYIGPVDGHNIDDLIAILKEVKGTKTTGPVLIHVVTEKGRGYPYAEKAADKYHGVTKFDPATGKQFKGSAITQSYTTYFAEALIAEAEVDKDIVAIHAAMGGGTGLNLFLRRFPTRCFDVGIAEQHAVTFAAGLACEGLKPFCAIYSSFMQRAYDQVVHDVDLQKLPVRFAMDRAGLVGADGPTHCGAFDVTFMACLPNMVVMAPSDEAELFHMVATAAAIDDRPSCFRYPRGNGVGVQLPPGNKGIPLEVGKGRILIEGERVALLGYGTAVQSCLAAASLVEPHGLLITVADARFCKPLDHTLIRSLAKPHEVLITVEEGSIGGFGSHVAHFLALDGLLDGKLKWRPLVLPDRYIDHGSPSVQLIEAGLTPSHVAATVLNILGNKREALQIMSS	NCBI
Hevea brasiliensis	*HbDXS2*		ABF18929.1	MAVSSSSFVSNQSFSPFLKAPRSNLCGRKQFCLRASAGHPDEEGKMMIRKEKDGWKIDFSGEKPPTPLLDTINYPVHTKNLSTQDLEQLAAELRADIVYSVSKTGGHLSSSLGVVELAVALHHVFSTPDDKIIWDVGHQAYPHKILTGRRSRMHTIRKTSGLAGFPKRDESVYDAFGAGHSSTSISAGLGMAVARDLLGKNNNVISVIGDGAMTAGQAYEAMNNAGFLDANLIVILNDNKQVSLPTATLDGPATPVGALSSALAKIQASTQFRKLREAAKSITKQIGGKTHQVAAKVDEYARGMISASGSTLFEELGLYYIGPVDGHNIEDLVTIFQKVKAMPAPGPVLIHIVTEKGKGYPPAEAAADKMHGVVKFDVQTGKQFKPKSPTLSYTQYFAEALIKEAETDNKIVAIHAAMGGGTGLNYFQKRFPDRCFDVGIAEQHAVTFAAGLATEGLKPFCAIYSSFLQRGYDQVVHDVDLQKLPVRFAMDRAGLVGADGPTHCGAFDIAYMACLPNMVVMAPSDEAELMHMVATAAAIDDRPSCFRFPRGNGIGAALPPNNKGTPLEIGKGRILMEGNRVAILGYGSIVQQCVEAASMLRTQGISVTVADARFCKPLDTDLIRQLAKEHEFLITVEEGSIGGFSSHVSHFLSLSGILDGPLKLRAMVLPDRYIDHGSPQDQIQEAGISSNHITATVLSLLGKPKEALQFKR	NCBI
Medicago truncatula	*MtDXS1*		CAD22530.1	MDLCSLACPSFVTPCDPRRTLPLPSSSSSHSQWGLHFFSHSQQHNKLKKKFGVVHASLSEMGEYYSQRPPTPLLDTINYPIHMKNLSTKELKQLADELRSDVIFSVSRTGGHLGSSLGVVELTIALHYVFNTPQDKILWDVGHQSYPHKILTGRRDKMHTMRQTNGLSGFTKRSESEYDSFGTGHSSTTISAGLGMAVGRDLKGRKNDVVAVIGDGAMTAGQAYEAMNNAGYLDSDMIVILNDNKQVSLPTATLDGPIPPVGALSSALSRLQSNKPLRELREVAKGVTKRIGGPMHELAAKVDEYARGMISGTGSTLFEELGLYYIGPVDGHNIDDLVAILKEVKTTNSTGPVLIHVITEKGRGYPYAQKAADKYHGVAKFDPP	NCBI

Species	Gene name	Gene ID	Gene Bank	Protein sequence	Database
				TGKQFKVAAKTQSYTTYFAEALIAEAKADKDIIAIHAAMGGGTGMNIFHRRFPTRCFDVGIAEQHAVTFAAGLACEGLKPFCAIYSSFLQRAYDQVVHDVDLQKLPVRFAMDRAGLVGSDGPTHSGSFDVTFMACLPNMVVMAPSDEAELCHMVATAAAIDDRPSCFRYPRGNGIGVELPTEYKGIPLEIGKGRILIEGERVALLGYGSAVQNCLAAASLVEQHGLRLTVADARFCKPLDRSLIRSLAKSHEVLITVEEGSIGGFGSHVAQFMALDGLLDGNLKWRPVVLPDRYIDHGSPADQLCMAGLTPSHIAATVFNILGQTREALEVMS	
Medicago truncatula	*MtDXS2*		CAN89181.1	MALSSCLLKPNHSLLQCHKFIAPNPNHGYRNQFCVMASSNSDEERTVIRKEKDEWKINFSAEKPPTPLLDTVNFPVHMKNLTTEDLEQLAAELRADIVHSVSDTGGHLSSSLGVVELSVALHHVFDTPDDKIIWDVGHQAYPHKILTGRRSRMHTIRKTSGLAGFPKRDESVHDAFGVGHSSTSISAGLGMAIARDLLGKKNSVISVIGDGAMTAGQAYEALNNAGFLDSNLIVILNDNKQVSLPTATLDGPATPVGALSSTLSKIQASRKFRKLREATKNITKQIGGQTHLVASKVDKYARDFISGSGSSLFEELGMYYIGPMDGHNIEDLVNIFEKVKATPASGPVLIHIVTEKGKGYPPAVAAADRMHGVVKFDPKTGHQFKPKPSTLAYTQYFADSLIKEAEMDNKIVAIHAAMGGGTGLNYFQKRFPDRCFDVGIAEQHAVTFAAGLATEGLKPFCAIYSTFLQRGYDQVVHDVDLQKLPVRFAMDRAGLVGADGPTHCGAFDITFMACLPNMIVMAPSDEAELMNMVATAAAIDDRPSCFRFPRGNGIGADLPLHNKGTPLEIGKGRILLEGSRVAILGYGCMVQQCMKAAEMLRAVGVYVTVSDARFCKPLDTDLIRLLAREHEILITVEEGSIGGFGSHVSQFLSLAGLLDGPLKLRSMMLPDRYIDHGAPNDQIDEAGLSSKHILATVLSLLEMPKEALFF	NCBI
Oryza sativa	*OsDXS1*	LOC_Os05g33840	NP_001055524	MALTTFSISRGGFVGALPQEGHFAPAAAELSLHKLQSRPHKARRRSSSISASLSTEREAAEYHSQRPPTPLLDTVNYPIHMKNLSLKELQQLADELRSDVIFHVSKTGGHLGSSLGVVELTVALHYVFNTPQDKILWDVGHQSYPHKILTGRRDKMPTMRQTNGLSGFTKRSESEYDSFGTGHSSTTISAALGMAVGRDLKGGKNNVVAVIGDGAMTAGQAYEAMNNAGYLDSDMIVILNDNKQVSLPTATLDGPAPPVGALSSALSKLQSSRPLRELREVAKGVTKQIGGSVHELAAKVDEYARGMISGSGSTLFEELGLYYIGPVDGHNIDDLITILREVKSTKTTGPVLIHVVTEKGRGYPYAERAADKYHGVAKFDPATGKQFKSPAKTLSYTNYFAEALIAEAEQDNRVVAIHAAMGGGTGLNYFLRRFPNRCFDVGIAEQHAVTFAAGLACEGLKPFCAIYSSFLQRGYDQVVHDVDLQKLPVRFAMDRAGLVGADGPTHCGAFDVTYMACLPNMVVMAPSDEAELCHMVATAAAIDDRPSCFRYPRGNGIGVPLPPNYKGVPLEVGKGRVLLEGERVALLGYGSAVQYCLAAASLVERHGLKVTVADARFCKPLDQTLIRRLASSHEVLLTVEEGSIGGFGSHVAQFMALDGLLDGKLKWRPLVLPDRYIDHGSPADQLAEAGLTPSHIAATVFNVLGQAREALAIMTVPNAD	NCBI

**Species**	**Gene name**	**Gene ID**	**Gene Bank**	**Protein sequence**	**Database**
Oryza sativa	*OsDXS2*	LOC_Os07g09190	NP_001059086	MALQASSSPSMFRAIPTNTNASCRRKLQVRASAAAAAANGGGDGKVMMRKEAASGAWKIDYSGEKPATPLLDTVNYPVHMKNLSTPELEQLAAELRAEIVHTVSKTGGHLSSSLGVVELAVALHHVFDTPEDKIIWDVGHQAYPHKILTGRRSRMHTIRQTSGLAGFPKRDESAHDAFGAGHSSTSISAALGMAVARDLLGKKNHVISVIGDGAMTAGQAYEAMNNSGYLDSNMIVVLNDNKQVSLPTATLDGPATPVGALSKALTKLQSSTKLRRLREAAKTVTKQIGGQAHEVAAKVDEYARGMVSASGSTLFEELGLYYIGPVDGHSVDDLVAIFNKVKSMPAPGPVLVHIVTEKGKGYPPAEAAADRMHGVVKFDPTTGRQFKSKCSTLSYTQYFAEALIREAEADDKVVGIHAAMGGGTGLNYFHKRFPERCFDVGIAEQHAVTFAAGLAAEGLKPFCAIYSSFLQRGYDQVVHDVDLQRLPVRFAMDRAGLVGADGPTHCGAFDVAYMACLPNMVVMAPADEAELMHMVATAAAIDDRPSCFRFPRGNGIGAVLPPNHKGTPLEVGKGRVLVGGNRVALLGYGTMVQACMKAAEALKEHGIYVTVADARFCKPLDTGLIRELAAEHEVLVTVEEGSIGGFGSHVAHYLSLSGLLDGPLKLRSMFLPDRYIDHGAPVDQLEEAGLTPRHIAATVLSLLGRPLEALQLS	NCBI
Oryza sativa	*OsDXS3*	LOC_Os06g05100	BAA83576	MFSLFVQRFNRRVAALPDVDDFFWEKDPTPILDTIDAPIHLKNLSSKELKQLAGEVRSEISFIMSRKCQPCNTGHSVVELAIAIHYVFNAPMDKILWDAGQHTYAHKILTGRRSLFHTIKQRKGLSGFTSRFESEYDPFGAGHGCNSLSAGLGMAVARDLGGRKNRIVTVISNWTTMAGQVYEAMGHAGFLDSNMVVILNDSRHTLLPKADSQSKMSINALSSALSKVQSSKGFRKFREAAKGLSKWFGKGMHEFAAKIDEYARGMIGPHGATLFEELGLYYIGPIDGNNIDDLICVLKEVSTLDSTGPVLVHVITENEKDSGGEFNSEITPDEEGPPDSSQDILKFLENGLSRTYNDCFVESLIAEAENDKHIVVVHGGMGIDRSIQLFQSRFPDRFFDLGIAEQHAVTFSAGLACGGLKPFCIIPSTFLQRAYDQIVEDVDMQKIPVRFAITSAGLVGSEGPTNSGPFDITFMSCLPNMIVMSPSNEDELIDMVATAAMVEDRPICFRYPKGAIVGTSGTLAYGNPLEIGKGEILAEGKEIAFLGYGDVVQRCLIARSLLFNFGIQATVANARFCKPLDIDLIRMLCQQHDFLITVEEGTVGGFGSHVSQFISLDGLLDGKIKVRIA	NCBI
Populus trichocarpa	*PtDXS1*		XP_002312717.1	MALSAFSLPAHVNSVTREVHVKKRPNGVCASLSESGEFPSQRPPTPLLDTVNYPIHMKNLSIKELKQLAEELRSDVIFNVSKTGGHLGSSLGVVELTVALHYVFNSPQDKILWDVGHQAYPHKILTGRRDKMHTIRQTNGLAGFTKRSESEYDCFGTGHSSTTISAGLGMAVGRDLKGRANNVVAVIGDGAMTAGQAYEAMNNAGYLDSDMIVILNDNKQVSLPTANLDGPIPPVGALSSALSRLQSNRPLRELREVAKGVTKQIGGPMHELAAKVDEYARGMISGSGSTLFEELGLYYIGPVDGHNIDDLIAILKEVKSTKTTGPVLIHVVTEKGRGYPYAERAADKYHGVNKFDPATGKQSKASASTQSYTTYFAEALIAEAEADKDVVAIHAAMGGGTGLNLFLRRFPTRCFDVGIAEQHAVTFAAGLACEGLKPFCAIYSFFLLILLYDNTQQYLVGIILLPDTDSSPHVDTIINVKMKLPVRFAMDRAGLVGADGPTHCGAFDVTYM	NCBI

**Species**	**Gene name**	**Gene ID**	**Gene Bank**	**Protein sequence**	**Database**
				ACLPNMVVMAPSDEAELFHMVATAAAIDDRPSCFRYPRGNGVGVQLPPENKGIPLEVGKGRILIEGERVALLGYGTAVQSCLAAASLVERHGIHLTVADARFCKPLDNALIRSLAKSHEVLITVEEGSIGGFGSHVAHFLALDGLLDGKLKWRPVVLPDRYIDHGSPADQLVEAGLTPSHIAATVFNILGQRRNSLEIMSS	
Populus trichocarpa	*PtDXS 2-1*		XP_002303416.1	MKIGSIFQFFLRASVSNSEDCEESKVVKKAEDGWKIDFSSGEKPSTPLLDTIDYPFHMDNLSTQDLEQLASELRADIVYSVAKTGGHLSSSLGVVELSVALHHVFNTPEDKIIWDVGHQAYPHKILTGRRSRMHTIRKTSGLAGFPKRDESVYDAFGAGHSSTSISAGLGMAVARDLLGKSNHVISVIGDGAMTAGQAYEAMNNAGFLDSNLIVILNDNKQVSLPTATLDGPATPVGALSSTLTKLQASAKFRKLHEAAKGITKQIDGQTHQVAAKVDEYARGMISASGSTLFEELGLYYIGPVDGHSIEDLVTIFQNVKAMPAPGPVLIHIITEKGKGYPPAEAAADKMHGVVKFDVKSGQQFKLKSSTLSYTRYFAESLIKEAEVDNKIVAIHAAMGGGTGLNYFQKRFPDRCFDVGIAEQHAVTFAAGLATEGLKPFCAIYSSFLQRGYDQVVHDVDLQKLPVRFAMDRAGLVGADGPTHCGAFDITYMACLPNMVVMAPSDEAELMHMVATAAAIDDRPSCFRFPRGNGIGTVLPPNNKGIALEIGKGRILMEGNRVAIMGYGSIVQQCAEAASMLRTQDISVTVADARFCKPLDTNLIRQLAKEHEILITVEEGSIGGFGSHVSHFLSSTGILDGPLKLRAMVLPDRYIDHGSPQDQIQEAGLSSNHITATVLSMLGKPKEALHEFK	NCBI
Populus trichocarpa	*PtDXS 2-2*		XP_002331678.1	LNFTGNKPSTPVLDTINHPIHMKNLSVQELDNLVDELREEIVYTVSKTGGHLSSSLGVAELTVALHHVFNTPEDKIIWDVGHQTYPHKILTGRRSRMHTIRQTFGLAGFPKREESEHDAFGAGHSSTSISAALGMAVGRDLLGKDNHVIAVIGDGAMTAGQAYEAMNNAGYLDSNLIIILNDNRQVSLPTATVDGPAPPVGALSRALTRLHSSRKFRQLREAAKGITKQIGGQTQEIAAKVDSYMRGMTGASGACLFEELGLYYIGPVDGHNVEDLVDLLKKVKAMPAPGPVLIHVITEKGKGYTPAEVAADKMHGVVKFDTKTGKQLKSKSNTLSYTQYFAESLIAEAEKDDKIVAIHAAMGGGTGLNLFQKRFPYRCFDVGIAEQHAVTFAAGLATEGLKPFCAIYSSFLQRGYDQVVHDVDLQKLPVRFALDRAGLVGADGPTHCGAFDTTFMASLPNMVVMAPSDETELIHMVATAAAIDDRPSCLRYPRGNGIGSIIPPNNKGTPLEVGKGRVLREGSRVAILGYGTIVQSCMQAAKLLEETGISATVADARFCKPLDGELIRQLAQEHEVLITVEEGSIGGFSSHVSHFLSLNGLLDGNIKWRPMMLPDRYIDHGSQTDQIEEAGLSPKHIASTAMSLVGM	NCBI
Populus trichocarpa	*PtDXS 4*		XP_002308644.1	ALPDIDDIFSDLIATPLLDVVENPIHLKNLTIKELKLLASEIRSELSSIMSKTQNDLKASLAVVELTVAIHHVFHAPVDKILWDVGEQTYAHKILTGRRSLMHTLRQKDGLSGFTSRSESEYDPFGAGHGCNSISAGIGMAIARDIKGKRERIVTVIGNGTTMAGQVYEAMGNAGYLDTNMIVILNDSRHSLHPKIEEGSKTSITALSSTLSKLQSSKSFRRLREVAKGVTKRIGMHELAAKVDEYARGMMGPLGSTLFE	NCBI

Species	Gene name	Gene ID	Gene Bank	Protein sequence	Database
				ELGLYYIGPVDGHNIGELVCVLQEVSSLDSMGPVLIHVITEENQCTEYKQPSEAMENQQEGILSSFDSNELLYSMHARTYSDCFVEALIMEAEKDKDIVIVHAGMEMDPSFQLFRERFPDRFFDLGMAEQHAVTFSAGLSCGGLKPFCIIPSAFMQRAYDQVVHDVDRQRIPVRFVITSAGLVGSDGPTMCGAFDITFMSCLPNMIVMAPSDEDELVDMVATAVHSDDHPICFRYPRGAIVGTDHYTRSGIPIEIGKGKILIEGKDVALLGYGEMVQNCLRARALLSKLGIEVTVADARFCKPLDMKLLRQLCENHAFLVTVEEGSIGGFGSHVSQFIALDGQLDGRTKWRPIVLPDKYIEHALPKEQLALAGLTGHHIAATVLRLLGRTREALLLM	
Ricinus communis	*RcDXS1*		XP_002516843.1	MALCAFSFPAHANKVTTASSDLQKSNYVSSNFLLKTDLLGQSMQKFNQGKKRPAGVCASLSETGEYHSQRPPTPLLDTINYPIHMKNLSIKELKQLSDELRSDVIFNVSITGGHLGSSLGVVELTVALHYVFNAPQDKILWDVGHQSYPHKILTGRRDKMQTMRQTNGLSGFTKRSESEYDCFGTGHSSTTISAGLGMAVGRDLKGRKNNVVAVIGDGAMTAGQAYEAMNNAGYLDSDMIVILNDNKQVSLPTANLDGPIPPVGALSSALSRLQSNKPLRELREVAKGVTKRIGGPMHELAAKVDEYARGMISGSGSTLFEELGLYYIGPVDGHNVDDLVAILKEVKSTKTTGPVLIHVVTEKGRGYPYAEKAADKYHGVTKFDPATGKQFKGSASTQSYTTYFAEALIAEAEVDKDVVAIHAAMGGGTGLNLFLRRFPTRCFDVGIAEQHAVTFAAGLACEGLKPFCAIYSSFMQRAYDQVVHDVDLQKLPVRFAMDRAGLVGADGPTHCGAFDVTFMACLPNMVVMAPSDEAELFHMVATAAAIDDRPSCFRYPRGNGIGVQLPPGNKGIPLEVGKGRILIEGERVALLGYGSAVQSCLAAASLMETYDLRITVADARFCKPLDHALIRSLAKSHEVLITVEEGSIGGFGSHVAHFLALDGLLDGKLKWRPVILPDRYIDHGSPADQLVEAGLTPSHIAATIFNILGNKREALQIMSA	NCBI
Ricinus communis	*RcDXS2*		XP_002533688.1	MAASSSVLRSTFLSTTTISSQDNHSLMRRPTTHVAAGKRKFNGVVAALENNASDDKNAMVMRDQQMKEKRTLNFSGTKPATPVLDTVNYPIHMNNLSVQDLEVLADELREEIVYVVSKTGGHLSSSLGVAELTVALHHVFNTPDDRIIWDVGHQAYPHKILTGRRSKMSSIRQTFGLAGFPKRDESEHDAFGAGHSSTSISAGLGMAVGRDLVGKNNHVIAVIGDGAMTAGQAYEAMNNAGYLDSNLIIILNDNKQVSLPTATVDGPAPPVGALSKALTKLQSSRKIRQLREAAKGITKQIGGQTHEIAAKVDTYVRGMAAGSGASLFEELGLYYIGPVDGHSVEDLVTILKKVKSLPAPGPVLIHVITEKGKGYSPAEVAADKMHGVVKFDPTSGKQLKSKSNTKSYTQYFAESLIAEAEQDDKIVAIHAAMGGGTGLNLFQKQFPEKCFDVGIAEQHAVTFAAGLATEGLKPFCAIYSSFLQRGYDQVVHDVDLQKLPVRFAIDRAGLVGADGPTHCGAFDVTYMACLPNMVVMAPSDETELMHMVATAAAIDDRPSCFRYPRGNGIGTILPPNNKGTPLEIGKGRILREGSRVALLGYGTMVQSCLAAADLLKKFNITATVADARFCKPLDGQLVRQLAQEHEILITVEEGSIGGFGSHVANFLCLNGLLDGNLKWRPMMLPDRYIDHGSQTDQIE	NCBI

Species	Gene name	Gene ID	Gene Bank	Protein sequence	Database
				EAGLSSKHIAATVVSLIGGQKDSLHLLNL?	
Ricinus communis	*RcDXS3*		XP_002514364.1	MGTASTQYPYGITAHSFAKFGHKLDTLSSSFPNKVGFSSVNLYQGSASITNSKGFVGRICSVPDLDDIFWEKVPTPILDVVENPIHLNNLTLQELKELADEIREELSSIMSRTQKAFKASLAVVELTVAIHHVFHAPVDKILWDVGEQTYAHKILTGRRSLMHTLRQKNGLSGFTSQSESEYDPFGAGHGCNSVSAGLGMAVARDMKGKRERVVTVISNGTTMAGQVYEAMSNAGYIDSNMIVILNDSRHSLHPKIEEGPKTSLNALSSTLSRLQSSKSFRKFREAAKGVTKRIGRGMHELAAKVDEYARGMIGPLGSTLFEELGLYYIGPVDGHNIEDLVCVLQEVASLDSMGPVLIHVVTEENRRRDNKQKIDTLENLQEGSSNSDPFLYSIHTRTYSDCFVEALIMEAEKDKDIVIVHAGMEMETAFQLIQERYPDRFFDVGMAEQHAVTFSAGLSCGGLKPFCIIPSTFLQRAYDQVVHDVDQQRIPVRFVITSAGLVGSDGPMQCGAFDITFMSCLPNMIVMAPSDEDELVDMVATAVQIDDHPVCFRYPRGAIVGTDHYMRIGIPIEIGKGKVLIEGKDVALLGYGAMVQNCLKARHLLSKLGIEVTVADARFCKPLDMKLLRQLCENHAFLVTVEEGSVGGFGSHVAQFLSLDGQLDGKVKWRPIVLPDTYIEHALPKEQLNLAGLTGHHIAATVLRLLGRTREALLLMC	NCBI
Toona ciliata	*TcDXS1*	Tci11G005020.1		MAFSSGFSIGTNQSIFPFLIASRPNLSCRKQFCLRASADNSDGEEAKKVTIKKEKGGWKIDFSGEKPATPLLDTVNYPIHMKNLCKQDLEQLAAELRADIVHSVSKTGGHLGSSLGVVEIAVALHHVFNTPEDRIIWDVGHQAYPHKILTGRRSKMHTIRKTSGLAGFPKRDESVHDAFGAGHSSTSISAGLGMAVARDLLGKKNNVISVIGDGALTAGQAYEAMNNAGFLDANLIVVLNDNKQVSLPTATLDGPASPVGALSSTLTKLQASTEFRKFRETAKRITKKIGGSTHEFAAKFDEYARGMISASGSTLFEELGLYYIGPVDGHKVEDLVTIFEKVKAMPAPGPVLIHVVTEKGKGYPPAEAAADKMHGVVKFDPKTGQQFKSKSPTLSYTQYFVDSLIKEAETDDKIVAIHAAMGGGTGLNYFLKRFPNRCFDVGIAEQHAVTFAAGLATEGLKPFCAIYSSFLQRGYDQVVHDVDLQKLPVRFAMDRAGLVGADGPTHCGAFDVTYMSCLPNMVVMAPSDEAELMHMVATAAAIDDRPSCFRFPRGNGIGAVLPPNNKGTPLEIGKGRVLIEGDRVAILGYGSIVQQCMEAASMLRSRGISVTVANARFCKPLDTDLIRQLAKEHEIIITVEEGSVGGFGSHVCHFLSLSGILDGPLKLRSMVLPDRYIDHGSPIDQIEEAGLSSRHISATVLSLLGRQKEALLLK?	NCBI
Toona ciliata	*TcDXS2*	Tci12G005860.1		MALSSGFSIVTNQSLFPFLTASRQNLSCRKRFCLRASADNSYGEEAKKVMIKKEKDGWKIDFSGEKPATPLLDTINYPVHMKNLSKQDLEQLAAELRADIVHSVSKTGGHLGSSLGVVEITVALHHVFNTPEDKIIWDVGHQAYPHKILTGRRSRMHTVRKTSGLAGFPKRDESTHDAFGAGHSSTSISAGLGMAVARELLGKKNNVISVIGDGALTAGQAYEAMNNAGFLDANLIVVLNDNKQVSLPTATLDGPASPVGALSSSLTKLQASTEFRKFREAAKSITKQIGGSTHEVAAKFDEYARGMISASGSTLFEELG	NCBI

**Species**	**Gene name**	**Gene ID**	**Gene Bank**	**Protein sequence**	**Database**
				LYYIGPVDGHNVEDLVTIFEKVKAMPTPGPVLIHVVTEKGKGYPPAEAAADKMHGVVKFDPKT<br>GQQFKSKSPTLPYTRYFVESLIKEAETDDKIVAIHAAMGGGTGLNYFQKRFPNRCFDVGIAEQH<br>AITFAAGLATEGLKPFCAIYSSFLQRGYDQVVHDVDLQKLPVRFAMDRAGLVGADGPTHCGAF<br>DITYMSCLPNMVVMAPADEAELMHMVATAAAIDDRPSCFRFPRGNGIGVVLPPSYKGTPLEIGK<br>GRVLTEGDRVAILGYGSIVQQCVEAASMLRSHDISVTVADARFCKPLDTDLIRQLAKDHEILITV<br>EEGAVGGFCSHVCHFLSLSGLLDGPLKLRSMVLPDRYIDHGSPVDQIEEAGLSSRHISATVLSLL<br>GRQKEALLLK	
Toona ciliata	*TcDXS 3*	Tci2 1G00 2650 .1		MALCAFSFPAHVNRASTVLDPQKSTSHTSHVFGGADLVSHSLHKLNQVRKRPGGVCASLSERE<br>EYHSQRPATPLLDTINYPIHMKNLSVKELKQLADELRSDVIFNVSKTGGHLGSSLGVVELTVALH<br>YVFNTPKDKILWDVGHQSYPHKILTGRRHMMHTMRQTNGLSGFTKRSESEHDCFGTGHSSTSIS<br>AGLGMAVGRDLKGRKNHVVAVIGDGAMTAGQAYEAMNNAGYLDSDMIVILNDNKQVSLPTA<br>TLDGPIQPVGALSSALSRLQSNRPLRELREVAKGVTKQIGGPMHELAAKVDEYARGMISGSRST<br>LFEELGLYYIGPVDGHSIDDLVAILEEVKSTKTTGPVLIHVVTEKGRGYPYAEKAADKYHGVAK<br>FDPATGKQFKPNAITQSYTTYFAEALIAEAEVDKDIVAIHAAMGGGTGLNLFLRRFPNRCFDVGI<br>AEQHAVTFAAGLASEGLKPFCAIYSSFMQRAYDQVVHDVDLQKLPVRFAMDRAGLVGADGPT<br>HCGSFDVTFMACLPNMVVMAPSDEAELFHMVATAAAINDRPSCFRYPRGNGIGVQLPPGNKGI<br>PLEVGKGRMLIEGERVALLGYGAAVQSCLAAASLLESHGLRLTVADARFCKPLDHGLIRSLAKS<br>HEVLITVEEGSIGGFGSHVAQFLALDGLLDGTVKWRPLVLPDQYIDHGSPAYQLAQAGLTPSHI<br>AATIFNILGQTREALEIMSYKK	NCBI
Toona ciliata	*TcDXS 4*	Tci2 2G00 9550 .1		MALCAFSFPARVNRASTALDPPKYTSLTSHVFWGADLVSHSLHKLNQVKKRPGGVCASLSERG<br>EYHSQRPATPLLDTINYPIHMKNLSVKELKQLADELRSDVIFNVSKTGGHLGSSLGVVELTVALH<br>YVFNTPKDKIVWDVGHQSYPHKILTGRRDKMHTMRQTNGLSGFTKRSESEHDCFGTGHSSTSIS<br>AGLGMAVGRDLKGRKNHVVAVIGDGAMTAGQAYEAMNNAGYLDSDMIVILNDNKQVSLPTA<br>TLDGPIPPVGALSSALSRLQSNRPLRELREVAKGVTKQIGGPMHELAAKVDEYARGMISGSRSTL<br>FEELGLYYIGPVDGHNIDDLVSILEEVKSTKTTGPVLIHVVTEKGRGYPYAEKAADKYHGVAKF<br>DPATGKQFKPSVITQSYTTYFAEALIAEAEVDKDIVAIHAAMGGGTGLNLFLRRFPNRCFDVGIA<br>EQHAVTFAAGLACEGVKPFCAIYSSFMQRAYDQVVHDVDLQKLPVRFAMDRAGLVGADGPTH<br>CGSFDVTFMACLPNMVVMAPSDEAELFHMVATAAAINDRPSCFRYPRGNGIGVQLPPGNKGIPL<br>EVGKGRVLIEGERVALLGYGSAVQSCLAAASLLESHGLRLTVADARFCKPLDYGLIRSLAKSHE<br>VLITVEEGSIGGFGSHVAQFLALDGLLDGTVKWSPLVLPDQYIDHGSPAYQLAQAGLTPSHIAAT	NCBI

Species	Gene name	Gene ID	Gene Bank	Protein sequence	Database
				VFNILGQTREALEIMSYKK	
Toona ciliata	*TcDXS5*	Tci25G002430.1		MASPFPKASFTSLLHAHPSSSKTSFPTTSFPSSDPKLKRVRASLDSTSTSDIKEFKLKGSRQIPEQN<br>GQIIKRILDFSGRKPSTPMLDTVNYPFQMKNLSIQELEMLAADLREELVYSVSKTGGHLSSSLGV<br>TELSVALHHVFNCPEDKIIWDVGHQAYMHKMLTGRRSRMHTIRQTCGLAGFPKRDENVHDAF<br>GVGHSSTSISAGLGMAVGRDLLEKNNNVISVIGDGAMTAGMAYEALNNAGFLDTNLIIILNDNQ<br>QVSLPTATVDGPAPPTGAFSQALRRLHSDSGFRQLCQAAKDKMKQFGGKTHEISAKTNEFSRE<br>MEGGSGAWFFEELGINYIGPVDGHNLEDLVYILKQVKDMPAAGPRLIHVITEKGKGYAPAEAAP<br>DKMHGVVKFDPATGKQLKSKPKTLSYTQYFAESLITEAERDDKIVAIHAAMGGGTGLNLFQKR<br>FPDRCFDVGIAEQHAVTFAAGLAAEGLKPFCAIYSSFLQRGFDQVAHDVDLQKLPVKFAIDRAG<br>LVGADGPTHCGAFDTTFMACLPNMVVMAPSDETELMHMVATAATINDRPCCFRYPRGNGFGSI<br>IPLNNKGTPLKVGKGRTLREGNDVAILGYGTVVKSCLEAADLLQMLGISATVADARFCKPLDGE<br>LIGKLAQKHEILITVEEGSIGGFSSHVSNFLALNGLLDGKLKWRPMMLPDRYIEHGSQADQIEEA<br>GLSSKHIAGTVFSLLAGVRRDVPS	NCBI
Toona ciliata	*TcDXS6*	Tci25G013250.1		MVTPSTKYPLGFAAHCHGKLGHRNTEFLSSNFSHELEISRINLCPISGFSSAPKHVSLSRICSLPDF<br>DDFFWEKNSTPILDAAENPIRLKNLTIKELKQLADEIHTELSSIVSKTEKSLKASLAVVELTVALH<br>HVFHAPVDKILWDVGEQTYAHKILTGRRSLIRTLRQKDGISGYTSRSESAYDPFSAAHGCNSVSA<br>GLGMAVARDIKGQREHIVTVISNGTTMCGQVYEAMSNAGYLDSNMIVILNDSQHSLHPKIEEGP<br>KTSINALSSTLSRIQSSKSFRKFREVAKDVTKRIGSGMHEWAAKVDEYARGLIGPVGATLFEELG<br>LYYIGPVDGHNIEDLISVLQEVASLDSMGPVLVHVITEENRRAEDNQKSEVMGKQQEGSSNPISL<br>LFSNHSRTYNDCFVEALVMEAEKDKDIALVHAGMGMNSSLQLFQEKFPEKFFDVGMAEQHAV<br>TFSAGLSCGGLKPFCIVPSAFLQRGYDQVVNDVDQQRLPVRFVITSAGLVGSDGPTQCGAFDITF<br>MSCLPSMIVMAPSDEDELVNMVATAASINDRPICFRYPRGAIVRAEYPMCSGIPIEIGKGKVLAE<br>GKDVALLGYGAMVQNCLKARALLSKLGIEVTVADARFCKPLDIKLLRELCQNHAFLITVEEGSI<br>GGFGSHVSQFIALDGLLDGGIKWRPIVLPDNYIEHASPTEQLALAGLTGHHIAATALSLLGRTRE<br>ALLLMCA	NCBI
Toona sinensis	*TsDXS1*	Maker00019581		MSPQVCFTRLSYYKTRMLYPFLLQSSLPAFLSPSFLSLITMALSSGFSIGTNQFIFPFLLASRPNPSC<br>RKQFCLRASADNSDGEEAKKVTIKKEKGGWKIDFSGEKPATPLLDTVNYPIHMKNLSKQDLEQ<br>LAAELRADIVHSVSKTGGHLGSSLGVVEIAVALHHVFNTPEDKIIWDVGHQAYPHKILTGRRSK<br>MHTIRKTSGLAGFPKRDESVHDAFGAGHSSTSISAGLGMAVARDLLGKKNNVISVIGDGALTAG<br>QAYEAMNNAGFLDANLIVVLNDNKQVSLPTATLDGPASPVGALSSTLTKLQASTEFRTFREAAK	NCBI

**Species**	**Gene name**	**Gene ID**	**Gene Bank**	**Protein sequence**	**Database**
				VVHDVDLQKLPVRFAMDRAGLVGADGPTHCGAFDVTYMSCLPNMVVMAPSDEAELMHMVATAAAIDDRPSCFRFPRGNGIGAVLPLSNKGTPLEIGKGRVLIEGDRVAILGYGSIVQQCVEAASMLRSRGISVTVADARFCKPLDTDLIRQLVKEHEILITVEEGSVGGFGSHVCHFLSLSGILDGPLKLRSMVLPDRYIDHGSPIDQIEEAGLSSTHISATILSLLGRQKEALLLK	
Toona sinensis	*TsDXS 2*	Maker000326 94		MALSSGFSIGTNQSVFPFLMASRQNLSCRKRFCLRASADNSYGEEAKKVMIKKGKDGWKIDFSGEKPATPLLDTINYPVHMKNLSKQDLEQLAAELRADIVHSVSKTGGHLGSSLGVVEITVALHHVFNTPEDKIIWDVGHQAYPHKILTGRRSRMHTVRKTSGLAGFPKRDESIHDAFGAGHSSTSISAGLGFLDANLIVVLNDNKQVSLPTATLDGPASPVGAISSTLTKLQASTEFRKIREAAKVVHDVDLQKLPVRFAMDRAGLVGADGPTHCGAFDITYMSCLPNMVVMAPADEAELMHMVATAAAIDDRPSCFRFPRGNGIGVVLPPNNKGTPLEIGKGRVLTEGDRVAILGYGSIVQQCVEAASMLRSRDISVTVADARFCKPLDTDLIRQLAKEHEILITVEEGAVGGFCSHVCHFLSLSGLLDGPLKLRSMVLPDRYIDHGSPVDQIEEAGLSSRHISATVLSLLGRQKEALLLKI	NCBI
Toona sinensis	*TsDXS 3*	Maker000053 41		MALCAFSFPAHVNRASTVLDPQKSTSHTSHVFGGADLVSHSHSLHKLNQVKKRPGGACASLSERGEYHSQRPVTPLLDTINYPIHMKNLSVKELKQLTDELRSDVIFNVSKTGGHLGSSLGVVELTVALHYRSESEHDCFGTGHSSTSISAGLGMAVGRDLKGRKNHVVAVIGDGAMTAGQAYEAMNNAGYLDSDMIVILNDNKQVSLPTATLDGPIPPVGALSSALSRLQSNRPLRELREVAKFDPATGKQFKPNAITKSYTTYFAEALIAEAEVDKDIVAIHAAMGGGTGLNLFLRRFPNRCFDVGIAEQHAVTFAAGLASEGLKPFCAIYSSFMQRAYDQVGKGRMLIEGERVALLGYGAAVQSCLAAASLLESHGLQLTVADARFCKPLDHGLIRSLAKSHEVLITVEEGSIGGFGSHVAQFLALDGLLDGTVKWRPLVLPDQYIDHGSPADQLAQAGLTPAHIAATIFNILGQTREALEIMSYKR	NCBI
Toona sinensis	*TsDXS 4*	Maker000281 22		MALCAFSFPAHVNRASTALDPLKYTSLTSHVFWGADLVSHSLHKLNQVKKRPGGACASLSEGGEYHSQRPATPLLDTINYPIHMKNLSVKELKQLADELRSDVIFNVSKTGGHLGSSLGVVELTVALHYVFNTPKDKIVWDVGHQSYPHKILTGRRDKMHTMRQTNGLSGFTKRSESEHDCFGTGHSSTSISAGLGMAVGRDLKGRKNHVVAVIGDGAMTAGQAYEAMNNAGYLDSDMIVILNDNKQVSLPTATLDGPIPPVGALSSALSRLQSNRPLRELREVAKVGKGRMLIEGERVALLGYGTAVQSCLAAASLLECHGLRLTVADARFCKPLDCGLIRNLAKSHEVLVTVEEGSIGGFGSHVAQFLALDGLLDGTVKWRPLVLPDQYIDHGSPAYQLAQAGLTPSHIAATVFNILGQTREALEIMSYKK	NCBI
Toona sinensis	*TsDXS 5*	Maker000113		MQHVSLSRICSLPDFDDFFWEKNSTPILDAVENPIRLKNLTIKELKQLADEIHTELSSIVSKTEKSLKASLAVVELTVALHHVFHAPVDKILWDVGEQTYAHKILTGRRSLIRTLRQKDGISGYTSRSESTYDPFSAAHGCNSVSAGLGMAVARDIKGKREHIVTVISNGTTMSGQVYEAMSNAGYLDSNMIVI	NCBI

Species	Gene name	Gene ID	Gene Bank	Protein sequence	Database
		52		LNDSRHSLHPKTEEGHQTSINALSSTLSRIQSSKSFRKFREVAKVVNDVDQQRLPVRFVITSAGLVGSDGPTQCGAFDITFMSCLPNMIVMAPSDEDELVNMVATAASINDRPVCFRYPRGAIVRAEYPMCSGIPIEIGKGKVLAEGKDVALLGYGAMVQNCLKARALLSKLGIEVTVADARFCKPLDIKLLRELCQNHAFLITVEEGSIGGFGSHVSQFIALDGLLDGGIKWRPIVLPDNYIEHASPTEQLALAGLTGHHIAATALSLLGRTREALLLMC	
Toona sinensis	*TsDXS6*	Maker00006994		MKRVRASIDSTSTSDIKEFILKGSRQIPEQNGQIIKRILDFSGRKPSTPILDTVNYPFQMKNLSIQELEMLAADLREEIVYSVSKTGGHLSSSLGVTELTVALHHVFNCPEDKIIWDVGHQAYMHKILTGRRSRMHTIRQTCGLAGFPKRDENVHDAFGVGHSSTSISAGLGMAVGRDLQEKNNHVISVIGDGAMTAGMAYEALNNAGFLGTNLIIILNDNQQVSLPTATVDGPAPPTGALSRTLRRLHSDSGFRELCQAAKDKMKQFGGKTHEISAKTNGFSREMEGSSGAWFFEELGINYIGPVDGHNLEDLVYILKQVKDMTAAGPILIHVITEKGKGYAPAEAAPDKMHGVVKFDPATGKQLKSKPKTLSYTQYFAESLIAEAERDDKIVAIHAAMGGGTGLNLFQKRFPDRCFDVGIAEQHAVTFAAGLAAEGLKPFCAIYSSFLQRGFDQVAHDVDLQKLPVKFAIDRAGLVGADGPTHCGAFDTTFMACLPNMVVMAPSDETELMHMVATAASINDRPCCFRYPRGNGFGSIIPLNNKGTPLKVGKGSTLREGNDVAILGYGTIVKSCLEAADLLKMLGISATWRPMMLPDRYIEHGSQADQIEEAGLNSKHIAGTLFSLLAGVRRDVLS	NCBI
Zea mays	*ZmDXS1*		NP_001157805.1	MALSTFSVPRGFLGVPAQDSHFASAVELHVNKLLQARPINLKPRRRPACVSASLSSEREAEYYSQRPPTPLLDTINYPVHMKNLSVKELRQLADELRSDVIFHVSKTGGHLGSSLGVVELTVALHYVFNAPQDRILWDVGHQSYPHKILTGRRDKMPTMRQTNGLAGFTKRAESEYDSFGTGHSSTTISAALGMAVGRDLKGGKNNVVAVIGDGAMTAGQAYEAMNNAGYLDSDMIVILNDNKQVSLPTATLDGPVPPVGALSSALSKLQSSRPLRELREVAKGVTKQIGGSVHELAAKVDEYARGMISGPGSSLFEELGLYYIGPVDGHNIDDLITILNDVKSTKTTGPVLIHVVTEKGRGYPYAERAADKYHGVAKFDPATGKQFKSPAKTLSYTNYFAEALIAEAEQDSKIVAIHAAMGGGTGLNYFLRRFPSRCFDVGIAEQHAVTFAAGLACEGLKPFCAIYSSFLQRGYDQVVHDVDLQKLPVRFAMDRAGLVGADGPTHCGAFDVAYMACLPNMVVMAPSDEAELCHMVATAAAIDDRPSCFRYPRGNGVGVPLPPNYKGTPLEVGKGRILLEGDRVALLGYGSAVQYCLTAASLVQRHGLKVTVADARFCKPLDHALIRSLAKSHEVLITVEEGSIGGFGSHIAQFMALDGLLDGKLKWRPLVLPDRYIDHGSPADQLAEAGLTPSHIAASVFNILGQNREALAIMAVPNA	NCBI
Zea mays	*ZmDXS2*		NP_001295426.1	MSPIMALQASSSSPSAFRAVPATANASCRRQFQVRAQVAGGSSSSSIGADGGKMMVSKEPAAAATSSGPWKIDFSGEKPPTPLLDTVNYPLHMKNLSILELEQLAAELRAEVVHTVSKTGGHLSSSLGVVELSVALHHVFDTPEDKIIWDVGHQAYPHKILTGRRSRMHTIRQTSGLAGFPKRDESAHDAFG	NCBI

Species	Gene name	Gene ID	Gene Bank	Protein sequence	Database
				VGHSSTSISAALGMAVARDLLGRKNHVISVIGDGAMTAGQAYEAMNNSGYLDANMIVVLNDN<br>KQVSLPTATLDGPSKPVGALSRALTKLQSSTKFRRLREAAKSVTKQIGGPTHEVAAKVDEYARG<br>MISASGSSLFEELGLYYIGPVDGHCVEDLVTIFEKVKSMPAPGPVLIHIVTEKGKGYPPAEAAAD<br>RMHGVVKFEPATGRQLKSKSPTLSYTQYFAESLIREAESDEKVVAIHAAMGGGTGLNYFQKRFP<br>ERCFDVGIAEQHAVTFAAGLAAEGLKPFCAIYSSFLQRGYDQVVHDVDLQRLPVRFALDRAGL<br>VGADGPTHCGAFDVAYMACLPNMVVMAPADEAELMHMVATAAAIDDRPSCFRFPRGNGVGA<br>ALPPGNKGVALEVGRGRVLVGGGTRVALLGYGAMVQACLKAAEALKEHDVYVTVADARFCK<br>PLDTALIRELAAEHEVLITAEEGSIGGFGSHVAHYLSLTGLLDGPLKLRSMFLPDRYIDHGAPQD<br>QIEEAGLTPRHIAATVLSLLGRPLEAMQLK	
Zea mays	*ZmDXS3*		NP_001170088.2	MDTAFLSPPLARNLVYDEFAVLHPTSYPFHTLRYLRCNPMYSRPLLTIAPASPSRGLIQRVAALP<br>DVDDFFWEKDPTPILDTIDAPIHLKNLSSKELKQLADEVRSEIAFIMSRKCQPCGADRSVVELTIA<br>IHYVFNAPMDKILWDAGQHAYAHKILTGRRSLFHTITQKNGLSGFTSRFESEYDPFGAGHGCNS<br>LSAGLGMAVARDINGRKNRIVTVISNWTTMAGQVYEAMGHAGFLDSNMVVILNDSCHTLLPK<br>ADGGAKMSVNAFSSALSKIQSSKGFRRFREAAKGLAKWFGKGMHEFAAKVDEYARGMIGPHG<br>ATLFEELGLYYIGPIDGRNIDDLICVLKEVASLDSTGPVLVHVITGTENDTGGIIGSEINAVEEGPS<br>NSSPDLLKFLGTGLSRTYNDCFVEALTAEAENDKRIVVVHGGMGIDRSLRLFQSRFPDRFFDLGI<br>AEQHAVTFSAGLACGGLKPFCIIPSTFLQRAYDQIIEDVDMQKIPVRFAITNAGLVGSEGPTNSGP<br>FDITFMSCLPNMIVMSPSNEDELIDMVATAAMIEDRPICFRYPRGAIVGTSGSVTYGNPFEIGKGE<br>ILVEGKEIAFLGYGEVVQRCLIARSLLSNFGIQATVANARFCKPLDIDLIRTLCQQHSFLITVEEGT<br>VGGFGSHVSQFISLDGLLDGRTKWRPIVLPDRYIEHASLAEQLDLAGLTAHHIAATALTLLGRHR<br>DALLLMK	NCBI

Table S3 CDS sequence of *TcDXSs* from *T. ciliata*

Gene name	CDS sequence
TcDXS1	ATGGCTTTTTCTTCTGGGTTTTCCATTGGAACGAACCAATCCATTTTTCCATTTCTTATAGCTTCTAGACCAAACCTGAGTTG<br>CAGAAAACAGTTCTGTCTGAGAGCCTCAGCTGATAACTCGGATGGGGAGGAAGCGAAGAAGGTGACGATAAAGAAAGAA<br>AAGGGTGGATGGAAAATTGATTTTTCCGGGGAAAAACCAGCCACACCATTGTTAGACACAGTAAATTACCCAATTCATATG<br>AAGAATCTATGTAAACAGGATCTTGAACAACTAGCAGCAGAACTTAGAGCAGATATTGTGCACAGTGTTTCAAAGACAGGT<br>GGGCATCTTGGTTCAAGCTTAGGTGTAGTGGAAATAGCAGTTGCATTGCATCATGTTTTCAATACACCTGAAGACAGAATTA

	TATGGGATGTCGGCCATCAGGCTTACCCCCACAAAATTCTGACAGGAAGAAGATCCAAGATGCATACCATAAGGAAAACTT<br>CAGGGCTCGCAGGATTTCCAAAAAGAGATGAGAGCGTTCATGATGCTTTTGGGGCAGGACATAGTTCCACAAGCATCTCTG<br>CTGGTCTTGGTATGGCAGTTGCAAGGGACCTTCTAGGGAAGAAAAATAATGTTATTTCTGTGATTGGAGATGGAGCCCTGA<br>CTGCAGGACAAGCATACGAGGCCATGAACAATGCAGGATTTCTCGACGCCAATCTTATTGTTGTGCTGAATGACAATAAGC<br>AAGTCTCTCTACCCACTGCTACTCTCGATGGTCCTGCGTCCCCAGTTGGAGCCCTCAGCAGTACATTAACCAAGCTCCAAG<br>CTAGCACTGAGTTCCGCAAATTTCGTGAAGCAGCAAAAAGAATCACGAAGCAAATTGGTGGATCAACACATGAATTTGCA<br>GCGAAATTCGATGAGTATGCAAGAGGAATGATCAGTGCTTCCGGGTCAACTCTCTTTGAGGAGCTAGGGTTATACTACATTG<br>GTCCAGTGGATGGACACAAAGTTGAAGATTTAGTAACCATCTTTGAAAAAGTGAAAGCAATGCCTGAACCAGGACCGGTT<br>CTGATCCATGTTGTAACAGAAAAAGGGAAGGGCTATCCCCCGGCAGAAGCAGCAGCTGATAAAATGCATGGAGTTGTCAA<br>GTTTGATCCAAAAACAGGCCAGCAATTTAAGTCCAAATCCCCTACGCTTTCATATACACGGTACTTTGTTGACTCTCTGATA<br>AAAGAAGCTGAGACGGATGATAAAATTGTAGCCATCCATGCCGCAATGGGAGGCGGTACAGGTCTCAATTATTTCCTGAAA<br>AGGTTTCCAAATCGCTGCTTTGACGTCGGAATTGCTGAGCAACATGCTGTTACTTTTGCAGCTGGTTTAGCTACAGAGGGT<br>CTCAGGCCATTTTGTGCCATCTACTCATCATTTCTGCAACGAGGATATGATCAAGTGGTACATGATGTAGATCTTCAAAAATT<br>ACCTGTCCGCTTTGCAATGGATCGAGCTGGTTTGGTTGGTGCAGATGGACCTACTCATTGTGGTGCATTTGATGTCACATAC<br>ATGTCTTGCTTGCCCAACATGGTGGTCATGGCTCCATCTGATGAAGCTGAGCTTATGCACATGGTTGCCACAGCAGCAGCTA<br>TAGATGACAGACCCAGCTGCTTCAGATTTCCAAGGGGAAATGGAATTGGAGCAGTTCTCCCACCTAATAACAAAGGAACC<br>CCACTTGAGATTGGAAAGGGAAGAGTACTAATAGAAGGCAATAGAGTTGCTATTTTGGGGTACGGTTCTATAGTTCAACAA<br>TGCATGGAGGCTGCAAGCATGCTCAGGAGCCGAGGCATATCTGTGACAGTGGCCGATGCAAGATTCTGCAAACCTTTGGAT<br>ACAGACCTCATTAGACAGTTGGCCAAAGAGCATGAAATCCTAATAACTGTGGAAGAGGGTTCTGTTGGAGGCTTTGGCTCT<br>CATGTATGTCACTTCCTAAGCTTAAGTGGTATTCTGGATGGACCTCTCAAGTTGAGATCTATGGTGCTTCCTGATAGATATATT<br>GACCATGGATCACCCATAGATCAAATTGAAGAAGCAGGGCTATCCTCAAGGCATATCTCTGCAACAGTCTTATCTCTCTTAG<br>GGAGGCAAAAAGAAGCCCTTCTGCTTAAGTAA
TcDXS2	ATGGCTCTTTCTTCTGGGTTTTCTATTGTAACAAACCAATCCCTTTTTCCATTTCTTACGGCTTCTAGACAGAACCTGAGTTG<br>CAGAAAAAGGTTCTGTTTGAGAGCCTCGGCTGATAACTCATATGGGGAGGAAGCGAAGAAGGTGATGATAAAGAAAGAA<br>AAGGATGGATGGAAAATTGATTTTTCCGGGGAAAAACCTGCCACACCATTGTTAGACACAATAAATTACCCAGTTCATATG<br>AAGAATCTATCTAAACAGGATCTTGAACAACTAGCAGCAGAGCTTAGAGCAGATATTGTGCACAGTGTATCAAAGACAGGT<br>GGGCATCTTGGTTCAAGCTTAGGAGTGGTGGAGATAACAGTAGCATTGCATCATGTTTTCAACACACCTGAAGATAAAATTA<br>TATGGGATGTTGGCCATCAGGCTTACCCCCACAAAATTCTGACAGGAAGAAGATCCAGGATGCATACTGTAAGGAAAACTT<br>CAGGGCTTGCCGGATTTCCTAAAAGAGATGAGAGTACTCATGATGCTTTTGGTGCAGGACATAGTTCCACAAGCATCTCTG<br>CTGGTCTTGGTATGGCAGTTGCAAGGGAACTTCTAGGGAAGAAAAATAATGTTATTTCTGTGATTGGAGATGGAGCCCTGA<br>CTGCAGGACAAGCATATGAGGCCATGAACAATGCAGGATTTCTAGACGCTAATCTAATTGTCGTGCTGAATGACAATAAGC<br>AAGTCTCTCTACCCACTGCTACTCTTGATGGTCCTGCATCTCCAGTTGGAGCCCTTAGCAGTTCTTTAACCAAGCTCCAAGC

	TAGCACTGAGTTCCGCAAATTTCGTGAAGCGGCAAAAAGCATCACTAAGCAAATTGGCGGATCAACACATGAAGTTGCAG<br>CAAAATTCGATGAGTATGCAAGAGGAATGATCAGCGCTTCCGGGTCAACTCTCTTTGAGGAGCTAGGGTTATACTACATAG<br>GTCCAGTGGATGGACACAATGTTGAAGATTTAGTAACCATCTTTGAAAAAGTGAAAGCAATGCCTACACCAGGGCCAGTTC<br>TGATCCATGTTGTAACAGAAAAAGGGAAGGGCTATCCCCCAGCAGAAGCAGCAGCTGATAAAATGCATGGAGTAGTCAAG<br>TTTGATCCCAAAACAGGCCAGCAATTTAAGTCCAAATCCCCCACGCTTCCATATACACGGTACTTCGTTGAATCTCTGATAA<br>AAGAAGCTGAGACGGATGATAAGATTGTTGCGATCCATGCCGCAATGGGAGGCGGCACTGGTCTCAATTATTTCCAGAAAA<br>GGTTTCCAAATCGATGCTTTGACGTGGGGATCGCTGAGCAACATGCTATTACTTTTGCAGCTGGTTTAGCTACAGAGGGTCT<br>CAAGCCATTTTGTGCCATCTACTCATCATTTCTGCAACGAGGATATGATCAGGTGGTACATGATGTAGATCTTCAAAAATTAC<br>CTGTCCGCTTTGCAATGGATCGAGCTGGTTTGGTTGGTGCAGATGGACCTACTCATTGTGGAGCATTCGATATAACATACAT<br>GTCTTGCTTGCCCAACATGGTGGTCATGGCTCCAGCTGATGAAGCTGAGCTTATGCACATGGTTGCCACAGCAGCAGCTAT<br>AGATGACAGACCCAGCTGCTTCAGATTTCCAAGGGGAAATGGAATTGGAGTAGTTCTCCCACCTAATTACAAAGGAACCCC<br>ACTTGAGATTGGGAAGGGAAGAGTACTAACAGAAGGTGATAGAGTTGCTATTTTGGGGTATGGTTCTATTGTTCAACAATG<br>TGTGGAAGCTGCAAGCATGCTAAGGAGCCATGACATATCTGTGACAGTGGCTGATGCAAGATTCTGCAAACCTTTGGATAC<br>AGACCTCATTAGACAGTTGGCCAAAGAGCATGAAATCCTAATTACTGTGGAAGAGGGTGCTGTTGGAGGCTTTTGCTCCCA<br>TGTATGTCACTTCTTAAGCTTAAGTGGTCTTCTGGATGGACCTCTCAAGTTGAGATCTATGGTGCTTCCTGATAGATACATTG<br>ACCATGGATCACCCGTAGATCAGATTGAAGAAGCAGGGCTATCCTCAAGGCATATCTCTGCAACAGTCTTATCTCTCTTAGG<br>GAGGCAAAAAGAAGCCCTTTTGCTTAAGTAG
TcDXS5	ATGGCTTCCCCTTTTCCCAAAGCAAGCTTTACATCACTGCTCCATGCTCATCCTTCTTCAAGCAAAACTAGTTTCCCCACTA<br>CTTCTTTCCCTAGCAGTGACCCCAAGTTGAAGAGAGTTAGAGCTTCCCTAGACAGTACTAGTACAAGTGATATAAAGGAAT<br>TCAAATTGAAGGGGAGCCGGCAGATACCAGAACAGAATGGACAGATAATTAAAAGAATACTCGATTTCTCTGGACGAAAG<br>CCATCCACGCCTATGCTAGATACTGTCAATTATCCATTTCAAATGAAAAATCTATCGATTCAGGAACTTGAGATGTTGGCTGC<br>GGATCTACGAGAAGAGTTAGTGTATTCGGTGTCGAAGACCGGTGGGCATCTAAGTTCTAGCCTGGGCGTGACTGAGCTCTC<br>GGTGGCTCTTCACCATGTATTCAACTGTCCTGAAGATAAAATCATTTGGGATGTTGGTCACCAGGCATATATGCATAAGATGT<br>TAACAGGAAGGAGATCGAGAATGCACACAATTCGGCAGACCTGTGGGCTGGCTGGTTTTCCCAAGAGGGATGAGAATGTG<br>CACGATGCCTTCGGTGTTGGCCACAGTTCTACTAGCATTTCTGCTGGCTTGGGAATGGCAGTTGGAAGAGATTTGCTAGAG<br>AAGAACAACAATGTAATCTCTGTCATTGGCGATGGTGCTATGACAGCGGGAATGGCATACGAGGCATTGAACAATGCAGGT<br>TTTCTTGACACAAATCTTATCATCATCTTGAACGATAACCAACAAGTCTCCTTGCCCACCGCCACCGTGGACGGACCTGCTC<br>CGCCTACTGGGGCTTTCAGCCAAGCCTTAAGACGTCTACATTCTGATTCAGGATTTCGCCAACTATGCCAAGCTGCAAAGG<br>ACAAGATGAAGCAATTCGGAGGGAAAACGCATGAAATTTCAGCCAAAACCAATGAATTCTCGAGAGAAATGGAGGGTGG<br>TTCAGGGGCTTGGTTCTTTGAAGAACTAGGAATAAATTATATCGGCCCAGTTGATGGACATAACTTGGAAGACCTTGTTTAT<br>ATTTTAAAACAAGTCAAGGATATGCCAGCAGCAGGACCTAGACTTATTCATTTAATTACTGAAAAAGGCAAAGGCTATGCT<br>CCTGCTGAAGCTGCACCAGACAAGATGCATGGCGTGGTGAAATTCGATCCCGCGACAGGAAAACAGTTAAAGTCCAAACC

	AAAAACCCTTTCCTACACGCAGTATTTTGCAGAATCTCTTATAACCGAAGCCGAGAGGGATGATAAAATTGTTGCCATCCAT<br>GCAGCAATGGGTGGTGGTACTGGACTAAACTTATTTCAAAAGCGATTCCCTGATAGATGTTTCGATGTGGGCATAGCCGAA<br>CAACACGCTGTTACCTTTGCCGCTGGTTTGGCTGCTGAAGGATTGAAACCTTTTTGTGCAATTTACTCCAGCTTCTTACAAA<br>GAGGCTTTGATCAGGTAGCCCATGATGTGGACCTTCAAAAACTTCCGGTGAAATTCGCGATAGACAGGGCTGGCCTTGTTG<br>GGGCAGACGGTCCGACACATTGCGGGGCATTCGACACCACTTTCATGGCTTGTTTGCCCAACATGGTGGTCATGGCTCCGT<br>CAGATGAGACTGAGCTTATGCACATGGTTGCCACAGCTGCAACCATTAACGACCGGCCTTGTTGCTTTAGATATCCCAGAG<br>GAAATGGCTTCGGTTCAATCATTCCACTAAATAACAAAGGAACACCTTTAAAGGTTGGCAAGGGAAGGACTTTAAGGGAA<br>GGAAATGATGTGGCTATTTTAGGTTACGGAACAGTTGTGAAAAGCTGCTTGGAAGCAGCTGACCTTCTTCAAATGCTTGGA<br>ATTTCAGCAACTGTGGCTGATGCTCGATTCTGCAAGCCACTCGACGGAGAGCTAATAGGGAAACTAGCACAAAAGCACGA<br>GATCCTCATCACTGTCGAAGAAGGATCAATCGGAGGATTCAGCTCTCATGTTTCCAATTTCCTGGCTTTGAATGGATTATTA<br>GATGGAAAACTTAAGTGGCGGCCAATGATGCTTCCGGACAGATACATCGAGCATGGATCACAGGCTGATCAGATAGAAGA<br>GGCGGGGCTGAGTTCAAAGCATATTGCAGGAACTGTGTTTTCATTATTGGCAGGGGTACGCAGAGATGTGCCATCATAA

Table S2 Location of 8 provenances of *T. ciliata*

Number	Provenance	Latitude	Longitude	Altitude (m)
P1	Pupiao Yunnan	N25°04′	E99°06′	1513
P2	Puer Yunnan	N22°46′	E100°58′	1317
P3	Yongren Yunnan	N25°01′	E101°32′	1539
P4	Lechang Guangdong	N25°07′	E113°20′	359
P5	Wangmo Guizhou	N25°10′	E106°05′	500
P6	Xingyi Guizhou	N25°06′	E104°54′	1160
P7	Tianlin Guangxi	N24°17′	E106°13′	792
P8	Longlin Guangxi	N24°46′	E105°20′	624

Table S3 All primers were used in this study.

Primers were used to clone *TcDXSs* CDS sequence		
Primer name	Sequence（5'to3'）	
TcDXS1-F	ATGGCTTTTTCTTCTGGGTTT	
TcDXS1-R	TTACTTAAGCAGAAGGGCTTCT	
TcDXS2-F	ATGGCTCTTTCTTCTGGGTT	
TcDXS2-R	CTACTTAAGCAAAAGGGCTTCTT	
TcDXS5-F	ATGGCTTCCCCTTTTCCC	
TcDXS5-R	TTATGATGGCACATCTCTGCG	
Primers used to amplify CDS of *TcDXSs* for subcellular localization		
Primer name	Primer sequence (5’ to 3’)	
DXS1-GFP-F	atttggagaggacagggtaccATGGCTTTTTCTTCTGGGTTTTC	
DXS1-GFP-R	agtgtcgactctagaggatccCTTAAGCAGAAGGGCTTCTTTTTG	
DXS2-GFP-F	atttggagaggacagggtaccATGGCTCTTTCTTCTGGGTTTTC	
DXS2-GFP-R	agtgtcgactctagaggatccCTTAAGCAAAAGGGCTTCTTTTTG	
DXS5-GFP-F	atttggagaggacagggtaccATGGCTTCCCCTTTTCCCA	
DXS5-GFP-R	agtgtcgactctagaggatccTGATGGCACATCTCTGCGTACC	
Primers used for quantitative real time PCR		
Primer name	Sequence（5'to3'）	Amplified product length(bp)
TcDXS1-qPCR-F2	ACTTCAGGGCTCGCAGGATT	195
TcDXS1-qPCR-R2	GTTCATGGCCTCGTATGCTTG	
TcDXS2-qPCR-F1	ACAGAGGGTCTCAAGCCATTT	126
TcDXS2-qPCR-R1	CAAACCAGCTCGATCCATTGC	
TcDXS5-qPCR-F2	GGATTTCGCCAACTATGCCAA	171
TcDXS5-qPCR-R2	TCAAAGAACCAAGCCCCTGAA	

Table S4 Statistics of different motifs that bind to *TcDXSs* promoter

Motifs	Number					
	TcDXS1	*TcDXS2*	*TcDXS3*	*TcDXS4*	*TcDXS5*	*TcDXS6*
ABRE	2	0	5	3	3	2
TC-rich repeats	1	2	1	0	1	0
P-box	0	0	1	1	0	0
GARE-motif	0	0	4	2	0	0
TATC-box	0	0	0	1	0	0
LTR	0	0	0	0	1	1
CGTCA-motif	0	0	1	1	2	2
TGACG-motif	0	0	1	1	2	2
TCA-element	0	1	1	0	0	0
Box 4	12	9	6	6	8	9
TCT-motif	2	1	0	2	0	0
AE-box	1	0	1	0	0	0
CAG-motif	1	1	0	0	0	0
G-Box	1	0	3	0	3	1
ATC-motif	1	0	0	0	0	1
I-box	0	1	0	0	0	0
AT1-motif	0	2	0	0	0	1
chs-CMA1a	0	1	0	2	0	0
ACE	0	2	0	0	0	0
GATA-motif	0	0	1	0	2	1
TCCC-motif	0	0	0	1	1	0
GT1-motif	0	0	0	1	0	0
ATCT-motif	0	0	0	0	1	0
