# Supplementary figures and images for: Characterization of the 1-Deoxy-D-xylulose 5-Phosphate synthase Genes in Toona ciliata Suggests Their Role in Insect Defense
Source: Int J Mol Sci. 2023 Jan 25;24(3):2339. doi: 10.3390/ijms24032339 (PMC9917211; doi:10.3390/ijms24032339)

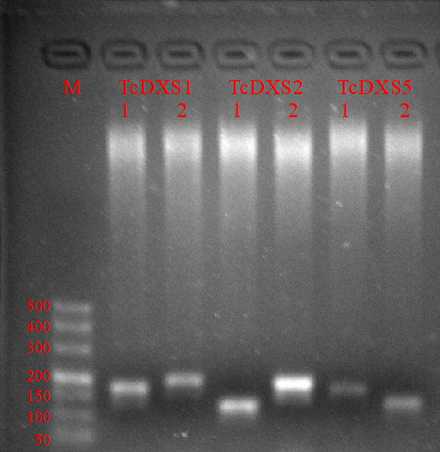

Supplement: Supplementary file 1 [file ijms-24-02339-s001.zip › Fig. S2 qPCR primer analysis of TcDXS1 TcDXS2 and TcDXS5 cDNA.jpg]

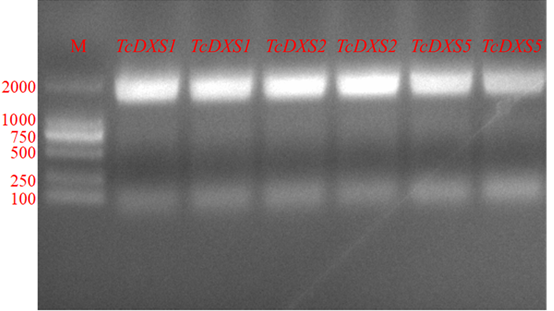

Supplement: Supplementary file 1 [file ijms-24-02339-s001.zip › Fig. S3 Amplification products of TcDXS1 TcDXS2 and TcDXS5 coding sequence.jpg]

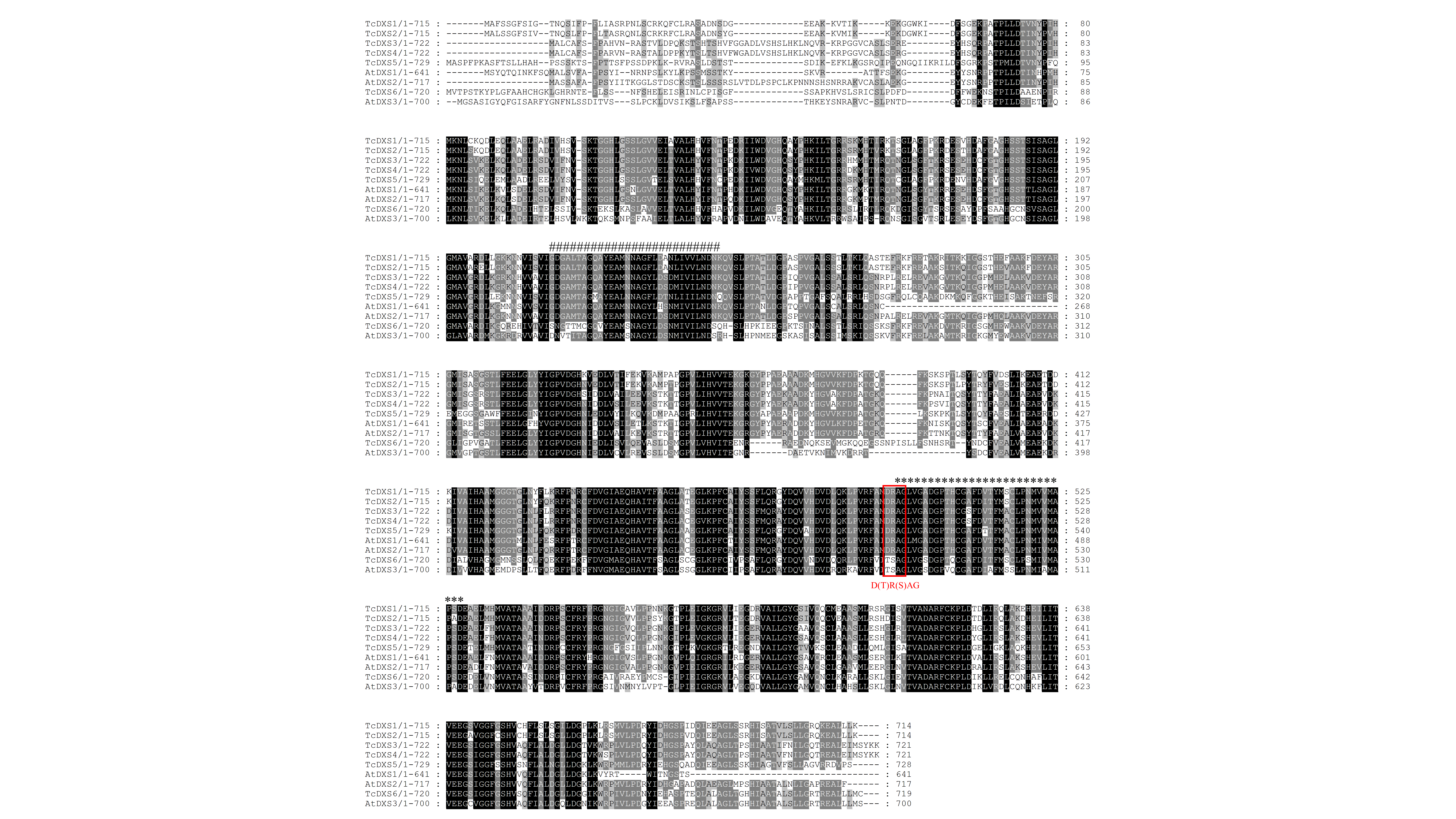

Supplement: Supplementary file 1 [file ijms-24-02339-s001.zip › Figure S1. Multiple sequence alignment.tif]
